# Crystal structures of hibiscus acid and hibiscus acid dimethyl ester isolated from *Hibiscus sabdariffa* (Malvaceae)

**DOI:** 10.1107/S2056989017011902

**Published:** 2017-08-21

**Authors:** Ahmed M. Zheoat, Alexander I. Gray, John O. Igoli, Alan R. Kennedy, Valerie A. Ferro

**Affiliations:** a Strathclyde Institute of Pharmacy and Biomedical Sciences, University of Strathclyde, 161 Cathedral Street, Glasgow G4 0RE, Scotland; b Westchem, Department of Pure & Applied Chemistry, University of Strathclyde, 295 Cathedral Street, Glasgow G1 1XL, Scotland

**Keywords:** crystal structure, natural products, hibiscus, lactone acids, hydrogen bonding

## Abstract

The biologically active title compounds have been isolated from *Hibiscus sabdariffa* plants, hibiscus acid as a dimethyl sulfoxide monosolvate [systematic name: (2*S*,3*R*)-3-hy­droxy-5-oxo-2,3,4,5-tetra­hydro­furan-2,3-di­carb­oxy­lic acid dimethyl sulfoxide monosolvate], C_6_H_6_O_7_·C_2_H_6_OS, (I), and hibiscus acid dimethyl ester [systematic name: dimethyl (2*S*,3*R*)-3-hy­droxy-5-oxo-2,3,4,5-tetra­hydro­furan-2,3-di­carboxyl­ate], C_8_H_10_O_7_, (II). Compound (I) forms a layered structure with alternating layers of lactone and solvent mol­ecules, that include a two-dimensional hydrogen-bonding construct. Compound (II) has two crystallographically independent and conformationally similar mol­ecules per asymmetric unit and forms a one-dimensional hydrogen-bonding construct. The known absolute configuration for both compounds has been confirmed.

## Abstract 2

The isolation and crystal structures of the title compounds from *Hibiscus sabdariffa* (Malvaceae) are described. Hibiscus acid dimethyl sulfoxide monosolvate forms a two-dimensional hydrogen-bonded motif, while hibiscus acid dimethyl ester (*Z*′ = 2) forms a one-dimensional hydrogen-bonded motif.

## Chemical context   

Lactone acid producing plants, including *Hibiscus sabdariffa* (Malvaceae), have been documented to have significant potential in the traditional treatment of various diseases. *H. sabdariffa* Linn is a species of hibiscus from the Malvaceae family, commonly known as ‘Karkade’ or ‘red sorrel’. It is used in traditional medicine in the form of herbal teas or cold drinks for its hypotensive and diuretic effects and to lower body temperature and blood viscosity (Ali *et al.*, 2005 ▸; Da-Costa-Rocha *et al.*, 2014 ▸). Little attention has been paid to organic acids from *H. sabdariffa*, specifically hibiscus acid. However, studies have documented the activity of hibiscus acid and hibiscus acid methyl ester. These report an inhibitory effect against enzymes, such as α-amylase and α-glucosidase (Hansawasdi *et al.*, 2000 ▸, 2001 ▸). As these compounds are not available commercially and to enable a study of their biological activities, we report on the extraction of hibiscus acid and hibiscus acid dimethyl ester from *H. sabdariffa* (Malvaceae), and on their purification and characterization. The crystal structures of the acid, as the dimethyl sulfoxide monosolvate, (I), and the diester, (II), are reported herein.

## Structural commentary   

**Figure 1:**
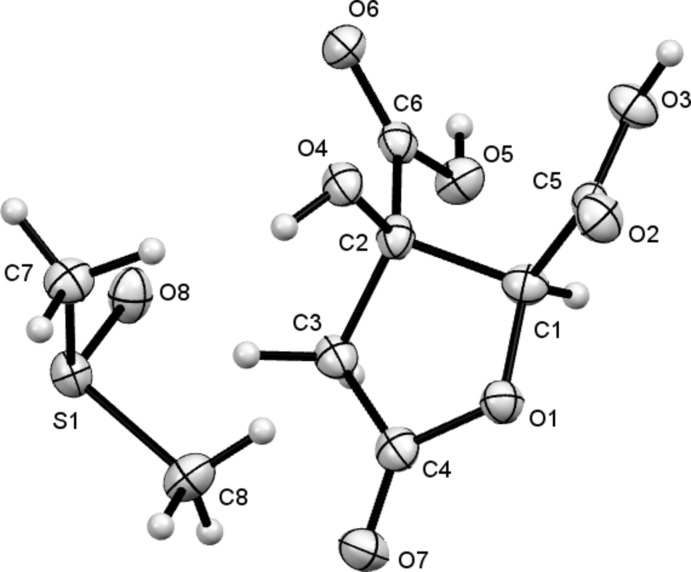
The mol­ecular structure of compound (I), with the atom labelling and 50% probability displacement ellipsoids.

The crystal structures of the 1:1 dimethyl sulfoxide (DMSO) solvate of hibiscus acid, (I), and of hibiscus acid dimethyl ester, (II), are shown in Figs. 1 ▸ and 2 ▸. The COO*R* (*R* = H or Me) groups lie in equatorial positions on their rings and the absolute configuration of both species is confirmed by the Flack parameter values (Parsons *et al.*, 2013 ▸), for arbitrarily named atoms in (I) [C2(*R*),C1(*S*), 0.00 (4)] and both arbitrarily named equivalent atoms in (II) [C3(*R*),C4(*S*) and C11(*R*),C12(*S*), 0.08 (17)] (Table 1 ▸). The absolute configuration found thus agrees with that originally proposed by Boll *et al.* (1969 ▸) for hibiscus acid. The structure of garcinia lactone, an epimer of hibiscus acid, has been reported (Mahapatra *et al.*, 2007 ▸). The comparable mol­ecular geometries of (I) and its epimer are similar. The five-membered ring of (I) adopts an envelope conformation, with the OH-bearing C2 atom 0.582 (6) Å out of the plane defined by the other four atoms.
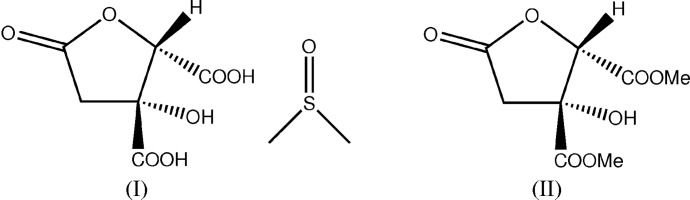


The structure of (II) contains two crystallographically independent mol­ecules (*A* and *B*) (*Z*′ = 2), whose mol­ecular geometries differ only by small deviations in torsion angles, for example, C3—C5—O5—C6 in *A* is 175.1 (4)°, whilst the equivalent angle in *B* (C11—C13—O12—C—14) is 180.0 (4)°. As with structure (I), the five-membered rings adopt envelope conformations, with the OH-bearing C atoms lying out of the plane of the other four atoms, here by 0.505 (5) and 0.530 (5) Å for mol­ecules *A* and *B*, respectively.

## Supra­molecular features   

**Figure 3:**
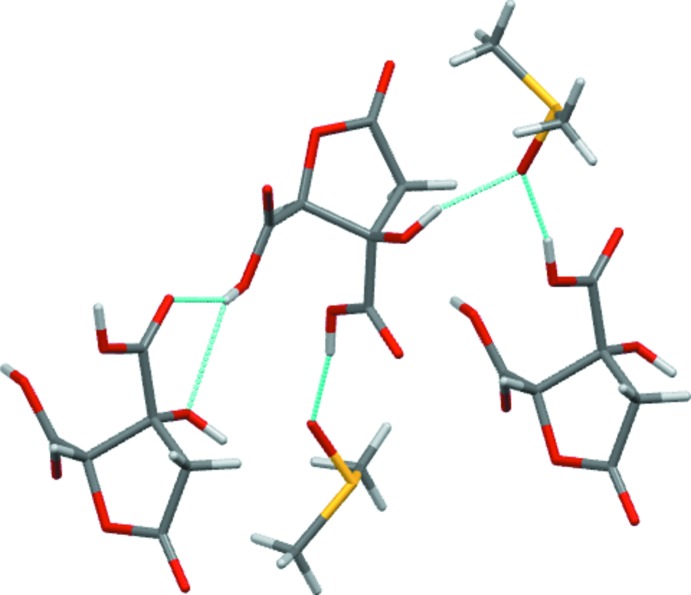
Hydrogen-bonding contacts in (I).

**Figure 4:**
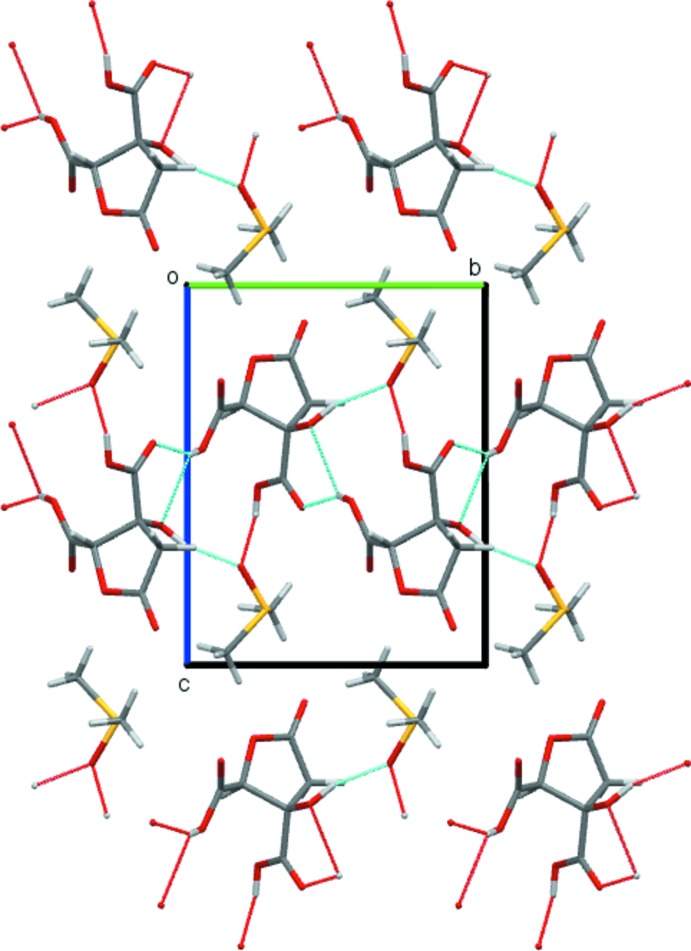
The crystal packing of compound (I), viewed along the *a* axis.

Despite containing two carb­oxy­lic acid functionalities, the structure of (I) does not feature the classic 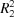
(8) carb­oxy­lic acid dimer motif. Instead, each of the three potential hydrogen-bond donors of the acid mol­ecule form inter­actions with a total of three separate neighbouring mol­ecules (Fig. 3 ▸). The H atom of the carb­oxy­lic acid group (O3—H) adjacent to the ether forms a bifurcated hydrogen bond that is accepted by the *R*OH and C=O functions (*i.e.* O4^i^ and O6^i^) of one neighbour, whilst the other two donors, the second carb­oxy­lic acid (O5—H) and the hy­droxy group (O4—H), form hydrogen bonds with atoms O8^ii^ and O8 of DMSO solvent mol­ecules, respectively (Table 2 ▸). These inter­actions combine to give a two-dimensional hydrogen-bonded layered structure, with DMSO and acid layers alternating along the *c*-cell direction (Fig. 4 ▸).

**Figure 5:**
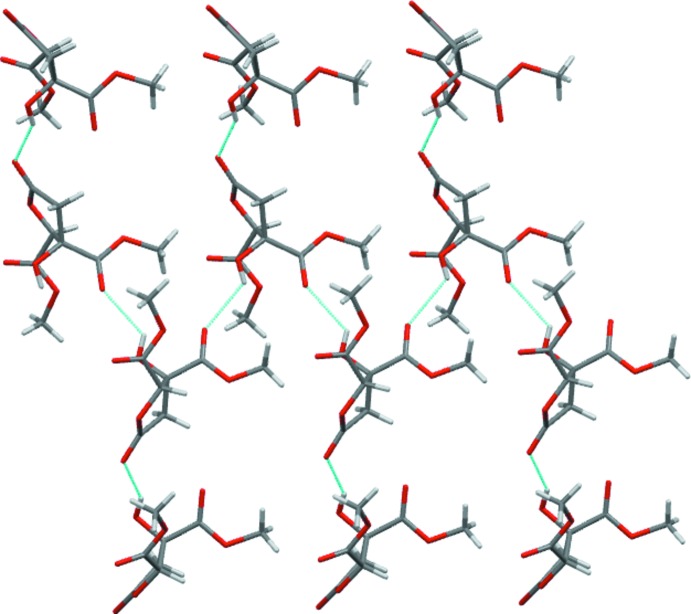
A section of the extended structure of (II), with the hydrogen-bonded polymer extending left and right parallel to the *a* axis.

Both independent mol­ecules in the structure of (II) donate single hydrogen bonds through their OH groups, but only one mol­ecule (*A*) acts as a hydrogen-bond acceptor (O3—H⋯O4^i^ and O10—H⋯O2^ii^; Table 3 ▸). That a total of four carbonyl O atoms do not act as acceptors is probably related to the low ratio of classic hydrogen-bond donors to acceptors in this compound. In (II), the hydrogen bonding combines to give a four-mol­ecule-wide one-dimensional ribbon of linked mol­ecules that propagates parallel to the *a* axis (Fig. 5 ▸).

## Database survey   

A search of the Cambridge Structural Database (Version 5.37, searched June 2017; Groom *et al.*, 2016 ▸) yielded few relevant structures. For hibiscus acid, only the structures of a Ca salt form (Glusker *et al.*, 1972 ▸) and of the diastereomer mentioned previously (Mahapatra *et al.*, 2007 ▸) have been reported. The closest relative of (II) to have been structurally described is a derivative with additional OH and Me substituents on the five-membered ring (Evans *et al.*, 1997 ▸).

## Synthesis and crystallization   

Dried *H. sabdariffa* calyces were crushed to a powder (500 g) and extracted in a Soxhlet apparatus using 2500 ml each of hexane, ethyl acetate and methanol. The methanol extract was dried and concentrated at 313 K by rotatory evaporation, yielding about 125 g (25%) of crude extract. The methanol extract (2 g) was dissolved in about 2 ml of methanol and subjected to gel filtration chromatography (GFC) using a glass column packed with a wet slurry of 30 g of Sephadex LH20 in methanol. Vials were collected (5 ml each) after elution with 100% methanol, which led to isolation of pure hibiscus acid (0.5%). Crystals of (I) were obtained by recrystallisation from DMSO. For nonsolvated material, ^1^H NMR [OC(CD_3_)_2_]: 5.31 (1H, *s*), 3.23 (1H, *d*, *J* = 17.19 Hz), 2.77 (1H, *d*, *J* = 17.18 Hz). HRMS: found 189.0000; calculated 189.0035.

Hibiscus acid dimethyl ester, (II), was obtained from the methanol extract (20 g) using vacuum liquid chromatography (VLC) eluted with solvent systems in different ratios to increase the polarity. The ethyl acetate portion was evaporated and a thick paste was obtained. A pure precipitate of the compound (5%) was obtained by addition of propan-2-ol to the dried ethyl acetate fraction. ^1^H NMR [OC(CD_3_)_2_]: 5.35 (1H, *s*), 3.23 (1H, *d*, *J* = 17.28 Hz), 2.77 (1H, *d*, *J* = 17.31 Hz), 3.87 (3H, *s*), 3.76 (3H, *s*). HRMS: found 218.000; calculated 218.035.

## Refinement   

Crystal data, data collection and structure refinement details are summarized in Table 1 ▸. For all structures, C-bound H atoms were placed in their expected geometrical positions and treated as riding, with C—H = 0.95–0.99 Å and *U*_iso_(H) = 1.5*U*_eq_(C) for methyl C atoms and 1.2*U*_eq_(C) for the other H atoms. The absolute configuraion was determined for the mol­ecules in both acid (I) for arbitrarily named atoms [C2(*R*),C1(*S*), Flack parameter 0.00 (4)] and both arbitrarily named equivalent atoms in (II) [C3(*R*),C4(*S*) (mol­ecule *A*) and C11(*R*),C12(*S*) (mol­ecule *B*), Flack parameter 0.08 (17)] (Parsons *et al.*, 2013 ▸).

## Supplementary Material

Crystal structure: contains datablock(s) I, II, global. DOI: 10.1107/S2056989017011902/zs2386sup1.cif

Structure factors: contains datablock(s) I. DOI: 10.1107/S2056989017011902/zs2386Isup2.hkl

Supporting information file. DOI: 10.1107/S2056989017011902/zs2386Isup4.cml

Structure factors: contains datablock(s) II. DOI: 10.1107/S2056989017011902/zs2386IIsup3.hkl

Supporting information file. DOI: 10.1107/S2056989017011902/zs2386IIsup5.cml

CCDC references: 1569231, 1569230

Additional supporting information:  crystallographic information; 3D view; checkCIF report

## Figures and Tables

**Figure 2:**
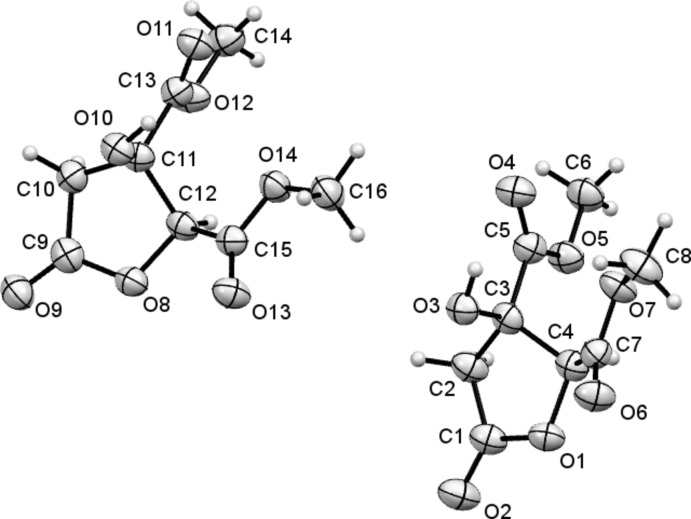
The mol­ecular structures of the two independent mol­ecules comprising the asymmetric unit of (II), with the atom labelling and 50% probability displacement ellipsoids.

**Table 1:** Experimental details

	(I)	(II)
Crystal data		
Chemical formula	C_6_H_6_O_7_·C_2_H_6_OS	C_8_H_10_O_7_
*M*_r_	268.24	218.16
Crystal system, space group	Monoclinic, *P*2_1_	Monoclinic, *P*2_1_
Temperature (K)	123	123
*a*, *b*, *c* (Å)	5.4258 (2), 8.9491 (3), 11.4365 (3)	9.3057 (6), 7.6934 (6), 13.4012 (11)
β (°)	94.092 (3)	96.243 (7)
*V* (Å^3^)	553.90 (3)	953.74 (12)
*Z*	2	4
Radiation type	Cu *K*α	Cu *K*α
μ (mm^−1^)	2.94	1.20
Crystal size (mm)	0.30 × 0.15 × 0.05	0.30 × 0.20 × 0.04

Data collection		
Diffractometer	Oxford Diffraction Gemini S CCD	Oxford Diffraction Gemini S CCD
Absorption correction	Multi-scan (*CrysAlis PRO*; Oxford Diffraction, 2010 ▸)	Multi-scan (*CrysAlis PRO*; Oxford Diffraction, 2010 ▸)
*T*_min_, *T*_max_	0.554, 1.000	0.747, 1.000
No. of measured, independent and observed [*I* > 2σ(*I*)] reflections	4397, 1854, 1640	8046, 3506, 2976
*R*_int_	0.054	0.036
(sin θ/λ)_max_ (Å^−1^)	0.619	0.622

Refinement		
*R*[*F* ^2^ > 2σ(*F* ^2^)], *wR*(*F* ^2^), *S*	0.047, 0.113, 1.05	0.044, 0.121, 1.10
No. of reflections	1854	3506
No. of parameters	169	281
No. of restraints	4	3
H-atom treatment	H atoms treated by a mixture of independent and constrained refinement	H atoms treated by a mixture of independent and constrained refinement
Δρ_max_, Δρ_min_ (e Å^−3^)	0.44, −0.25	0.23, −0.22
Absolute structure	Flack *x* determined using 698 quotients [(*I* ^+^) − (*I* ^−^)]/[(*I* ^+^) + (*I* ^−^)] (Parsons *et al.*, 2013 ▸)	Flack *x* determined using 1098 quotients [(*I* ^+^) − (*I* ^−^)]/[(*I* ^+^) + (*I* ^−^)] (Parsons *et al.*, 2013 ▸)
Absolute structure parameter	0.00 (4)	0.08 (17)

Computer programs: *CrysAlis PRO* (Oxford Diffraction, 2010 ▸), *SIR92* (Altomare *et al.*, 1993 ▸), *SHELXL2014* (Sheldrick, 2015 ▸) and *Mercury* (Macrae *et al.*, 2008 ▸).

**Table 2:** Hydrogen-bond geometry (Å, °) for (I)

*D*—H⋯*A*	*D*—H	H⋯*A*	*D*⋯*A*	*D*—H⋯*A*
O3—H1*H*⋯O4^i^	0.87 (2)	2.42 (4)	2.996 (4)	124 (3)
O3—H1*H*⋯O6^i^	0.87 (2)	1.98 (3)	2.805 (4)	158 (4)
O4—H3*H*⋯O8	0.87 (2)	1.87 (3)	2.714 (5)	160 (7)
O5—H2*H*⋯O8^ii^	0.89 (2)	1.73 (2)	2.603 (4)	167 (5)

Symmetry codes: (i) 
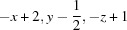
; (ii) 
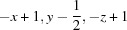
.

**Table 3:** Hydrogen-bond geometry (Å, °) for (II)

*D*—H⋯*A*	*D*—H	H⋯*A*	*D*⋯*A*	*D*—H⋯*A*
O3—H1*H*⋯O4^i^	0.88 (1)	2.36 (5)	2.951 (4)	125 (4)
O10—H2*H*⋯O2^ii^	0.88 (1)	2.03 (3)	2.802 (4)	147 (5)

Symmetry codes: (i) 
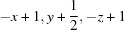
; (ii) 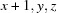.

## supplementary crystallographic information

### (2S,3R)-3-Hydroxy-5-oxo-2,3,4,5-tetrahydrofuran-2,3-dicarboxylic acid dimethyl sulfoxide monosolvate (I) Crystal data

**Table:**

C_6_H_6_O_7_·C_2_H_6_OS	*F*(000) = 280
*M**_r_* = 268.24	*D*_x_ = 1.608 Mg m^−^^3^
Monoclinic, *P*2_1_	Cu *K*α radiation, λ = 1.5418 Å
*a* = 5.4258 (2) Å	Cell parameters from 2057 reflections
*b* = 8.9491 (3) Å	θ = 6.3–72.8°
*c* = 11.4365 (3) Å	µ = 2.94 mm^−^^1^
β = 94.092 (3)°	*T* = 123 K
*V* = 553.90 (3) Å^3^	Fragment from a square plate, colourless
*Z* = 2	0.30 × 0.15 × 0.05 mm

### (2S,3R)-3-Hydroxy-5-oxo-2,3,4,5-tetrahydrofuran-2,3-dicarboxylic acid dimethyl sulfoxide monosolvate (I) Data collection

**Table:**

Oxford Diffraction Gemini S CCD diffractometer	1640 reflections with *I* > 2σ(*I*)
Radiation source: sealed tube	*R*_int_ = 0.054
ω scans	θ_max_ = 72.8°, θ_min_ = 3.9°
Absorption correction: multi-scan (CrysAlis PRO; Oxford Diffraction, 2010)	*h* = −6→6
*T*_min_ = 0.554, *T*_max_ = 1.000	*k* = −10→8
4397 measured reflections	*l* = −14→14
1854 independent reflections	

### (2S,3R)-3-Hydroxy-5-oxo-2,3,4,5-tetrahydrofuran-2,3-dicarboxylic acid dimethyl sulfoxide monosolvate (I) Refinement

**Table:**

Refinement on *F*^2^	Hydrogen site location: mixed
Least-squares matrix: full	H atoms treated by a mixture of independent and constrained refinement
*R*[*F*^2^ > 2σ(*F*^2^)] = 0.047	*w* = 1/[σ^2^(*F*_o_^2^) + (0.0678*P*)^2^] where *P* = (*F*_o_^2^ + 2*F*_c_^2^)/3
*wR*(*F*^2^) = 0.113	(Δ/σ)_max_ < 0.001
*S* = 1.05	Δρ_max_ = 0.44 e Å^−^^3^
1854 reflections	Δρ_min_ = −0.25 e Å^−^^3^
169 parameters	Absolute structure: Flack *x* determined using 698 quotients [(I+)-(I-)]/[(I+)+(I-)] (Parsons *et al.*, 2013)
4 restraints	Absolute structure parameter: 0.00 (4)

### (2S,3R)-3-Hydroxy-5-oxo-2,3,4,5-tetrahydrofuran-2,3-dicarboxylic acid dimethyl sulfoxide monosolvate (I) Special details

**Table:**

Geometry. All esds (except the esd in the dihedral angle between two l.s. planes) are estimated using the full covariance matrix. The cell esds are taken into account individually in the estimation of esds in distances, angles and torsion angles; correlations between esds in cell parameters are only used when they are defined by crystal symmetry. An approximate (isotropic) treatment of cell esds is used for estimating esds involving l.s. planes.
Refinement. Refined as a 2-component inversion twin

### (2S,3R)-3-Hydroxy-5-oxo-2,3,4,5-tetrahydrofuran-2,3-dicarboxylic acid dimethyl sulfoxide monosolvate (I) Fractional atomic coordinates and isotropic or equivalent isotropic displacement parameters (Å^2^)

**Table:**

	*x*	*y*	*z*	*U*_iso_*/*U*_eq_	
S1	0.90564 (19)	1.26444 (16)	0.85370 (9)	0.0235 (3)	
O1	0.6239 (6)	0.7220 (4)	0.8066 (3)	0.0233 (9)	
O2	1.0711 (6)	0.6125 (5)	0.7467 (3)	0.0285 (9)	
O3	0.9028 (6)	0.5501 (5)	0.5682 (3)	0.0276 (8)	
O4	0.8575 (6)	0.9112 (5)	0.6333 (3)	0.0232 (8)	
O5	0.3551 (6)	0.7366 (5)	0.4714 (3)	0.0265 (9)	
O6	0.6572 (6)	0.8844 (5)	0.4153 (3)	0.0254 (8)	
O7	0.4156 (6)	0.8927 (5)	0.9015 (3)	0.0300 (9)	
O8	0.8239 (7)	1.1798 (5)	0.7411 (3)	0.0295 (9)	
C1	0.6534 (8)	0.6840 (7)	0.6856 (4)	0.0231 (11)	
H1	0.5185	0.6149	0.6557	0.028*	
C2	0.6288 (8)	0.8370 (6)	0.6206 (4)	0.0219 (11)	
C3	0.4303 (8)	0.9097 (7)	0.6897 (4)	0.0236 (11)	
H3A	0.4466	1.0198	0.6901	0.028*	
H3B	0.2627	0.8823	0.6567	0.028*	
C4	0.4814 (8)	0.8461 (7)	0.8109 (4)	0.0244 (11)	
C5	0.9026 (8)	0.6125 (7)	0.6737 (4)	0.0221 (11)	
C6	0.5504 (8)	0.8209 (6)	0.4898 (4)	0.0216 (10)	
C7	1.2345 (8)	1.2784 (8)	0.8549 (4)	0.0273 (12)	
H7A	1.2788	1.3417	0.7897	0.041*	
H7B	1.3053	1.1786	0.8465	0.041*	
H7C	1.2997	1.3227	0.9292	0.041*	
C8	0.8906 (10)	1.1285 (8)	0.9679 (4)	0.0309 (13)	
H8A	0.9898	1.0411	0.9498	0.046*	
H8B	0.7184	1.0981	0.9739	0.046*	
H8C	0.9552	1.1720	1.0425	0.046*	
H2H	0.308 (11)	0.729 (8)	0.396 (3)	0.032 (17)*	
H1H	1.043 (7)	0.509 (7)	0.555 (5)	0.026 (15)*	
H3H	0.865 (17)	0.987 (7)	0.682 (6)	0.07 (3)*	

### (2S,3R)-3-Hydroxy-5-oxo-2,3,4,5-tetrahydrofuran-2,3-dicarboxylic acid dimethyl sulfoxide monosolvate (I) Atomic displacement parameters (Å^2^)

**Table:**

	*U*^11^	*U*^22^	*U*^33^	*U*^12^	*U*^13^	*U*^23^
S1	0.0241 (5)	0.0207 (7)	0.0254 (5)	0.0013 (5)	0.0002 (4)	−0.0021 (5)
O1	0.0228 (14)	0.024 (3)	0.0232 (15)	0.0022 (13)	0.0016 (11)	0.0007 (13)
O2	0.0237 (16)	0.030 (3)	0.0320 (17)	0.0050 (15)	0.0000 (13)	0.0007 (16)
O3	0.0213 (14)	0.032 (3)	0.0299 (16)	0.0032 (15)	0.0023 (12)	−0.0049 (16)
O4	0.0187 (14)	0.021 (2)	0.0299 (17)	−0.0025 (14)	0.0029 (12)	−0.0026 (15)
O5	0.0248 (14)	0.030 (3)	0.0243 (14)	−0.0046 (15)	−0.0009 (11)	0.0008 (14)
O6	0.0258 (15)	0.024 (2)	0.0267 (15)	−0.0024 (15)	0.0046 (12)	0.0010 (15)
O7	0.0308 (16)	0.033 (3)	0.0274 (17)	0.0032 (16)	0.0073 (13)	−0.0037 (17)
O8	0.0329 (18)	0.025 (3)	0.0296 (17)	0.0006 (17)	−0.0058 (14)	0.0030 (17)
C1	0.019 (2)	0.027 (3)	0.024 (2)	0.000 (2)	0.0022 (16)	−0.001 (2)
C2	0.0178 (19)	0.018 (3)	0.030 (2)	−0.0008 (19)	0.0023 (16)	0.001 (2)
C3	0.0184 (19)	0.023 (3)	0.030 (2)	−0.0014 (19)	0.0027 (16)	−0.003 (2)
C4	0.019 (2)	0.024 (3)	0.030 (2)	−0.0040 (19)	0.0025 (16)	−0.002 (2)
C5	0.023 (2)	0.017 (3)	0.028 (2)	0.0014 (19)	0.0073 (18)	0.005 (2)
C6	0.019 (2)	0.019 (3)	0.027 (2)	0.0031 (18)	0.0028 (16)	−0.0004 (19)
C7	0.0204 (18)	0.031 (4)	0.030 (2)	−0.001 (2)	0.0012 (16)	0.001 (2)
C8	0.032 (2)	0.033 (4)	0.027 (2)	−0.004 (2)	0.0027 (18)	0.006 (2)

### (2S,3R)-3-Hydroxy-5-oxo-2,3,4,5-tetrahydrofuran-2,3-dicarboxylic acid dimethyl sulfoxide monosolvate (I) Geometric parameters (Å, º)

**Table:**

S1—O8	1.532 (4)	C1—C5	1.511 (6)
S1—C7	1.788 (5)	C1—C2	1.559 (8)
S1—C8	1.791 (6)	C1—H1	1.0000
O1—C4	1.356 (7)	C2—C3	1.525 (6)
O1—C1	1.445 (6)	C2—C6	1.532 (6)
O2—C5	1.194 (6)	C3—C4	1.505 (7)
O3—C5	1.329 (6)	C3—H3A	0.9900
O3—H1H	0.87 (3)	C3—H3B	0.9900
O4—C2	1.406 (6)	C7—H7A	0.9800
O4—H3H	0.87 (3)	C7—H7B	0.9800
O5—C6	1.306 (6)	C7—H7C	0.9800
O5—H2H	0.89 (3)	C8—H8A	0.9800
O6—C6	1.206 (6)	C8—H8B	0.9800
O7—C4	1.195 (6)	C8—H8C	0.9800
			
O8—S1—C7	105.7 (2)	C2—C3—H3B	111.2
O8—S1—C8	104.6 (3)	H3A—C3—H3B	109.1
C7—S1—C8	98.0 (3)	O7—C4—O1	121.5 (5)
C4—O1—C1	109.3 (4)	O7—C4—C3	128.3 (5)
C5—O3—H1H	113 (4)	O1—C4—C3	110.1 (4)
C2—O4—H3H	116 (6)	O2—C5—O3	125.8 (5)
C6—O5—H2H	112 (4)	O2—C5—C1	125.6 (5)
O1—C1—C5	110.3 (4)	O3—C5—C1	108.6 (4)
O1—C1—C2	103.8 (4)	O6—C6—O5	125.7 (4)
C5—C1—C2	112.1 (4)	O6—C6—C2	122.1 (5)
O1—C1—H1	110.2	O5—C6—C2	112.1 (4)
C5—C1—H1	110.2	S1—C7—H7A	109.5
C2—C1—H1	110.2	S1—C7—H7B	109.5
O4—C2—C3	113.3 (4)	H7A—C7—H7B	109.5
O4—C2—C6	109.0 (4)	S1—C7—H7C	109.5
C3—C2—C6	112.9 (4)	H7A—C7—H7C	109.5
O4—C2—C1	108.7 (4)	H7B—C7—H7C	109.5
C3—C2—C1	99.6 (4)	S1—C8—H8A	109.5
C6—C2—C1	113.0 (5)	S1—C8—H8B	109.5
C4—C3—C2	103.0 (4)	H8A—C8—H8B	109.5
C4—C3—H3A	111.2	S1—C8—H8C	109.5
C2—C3—H3A	111.2	H8A—C8—H8C	109.5
C4—C3—H3B	111.2	H8B—C8—H8C	109.5
			
C4—O1—C1—C5	148.2 (4)	C2—C3—C4—O7	161.1 (5)
C4—O1—C1—C2	27.9 (5)	C2—C3—C4—O1	−17.9 (5)
O1—C1—C2—O4	82.0 (4)	O1—C1—C5—O2	−13.6 (8)
C5—C1—C2—O4	−37.1 (5)	C2—C1—C5—O2	101.5 (6)
O1—C1—C2—C3	−36.8 (4)	O1—C1—C5—O3	166.6 (4)
C5—C1—C2—C3	−155.9 (4)	C2—C1—C5—O3	−78.3 (6)
O1—C1—C2—C6	−156.8 (3)	O4—C2—C6—O6	−10.2 (7)
C5—C1—C2—C6	84.1 (5)	C3—C2—C6—O6	116.7 (5)
O4—C2—C3—C4	−83.0 (5)	C1—C2—C6—O6	−131.2 (5)
C6—C2—C3—C4	152.4 (5)	O4—C2—C6—O5	172.0 (4)
C1—C2—C3—C4	32.3 (5)	C3—C2—C6—O5	−61.2 (6)
C1—O1—C4—O7	174.3 (5)	C1—C2—C6—O5	50.9 (5)
C1—O1—C4—C3	−6.6 (5)		

### (2S,3R)-3-Hydroxy-5-oxo-2,3,4,5-tetrahydrofuran-2,3-dicarboxylic acid dimethyl sulfoxide monosolvate (I) Hydrogen-bond geometry (Å, º)

**Table:**

*D*—H···*A*	*D*—H	H···*A*	*D*···*A*	*D*—H···*A*
O3—H1*H*···O4^i^	0.87 (2)	2.42 (4)	2.996 (4)	124 (3)
O3—H1*H*···O6^i^	0.87 (2)	1.98 (3)	2.805 (4)	158 (4)
O4—H3*H*···O8	0.87 (2)	1.87 (3)	2.714 (5)	160 (7)
O5—H2*H*···O8^ii^	0.89 (2)	1.73 (2)	2.603 (4)	167 (5)

Symmetry codes: (i) −*x*+2, *y*−1/2, −*z*+1; (ii) −*x*+1, *y*−1/2, −*z*+1.

### Dimethyl (2S,3R)-3-Hydroxy-5-oxo-2,3,4,5-tetrahydrofuran-2,3-dicarboxylate (II) Crystal data

**Table:**

C_8_H_10_O_7_	*F*(000) = 456
*M**_r_* = 218.16	*D*_x_ = 1.519 Mg m^−^^3^
Monoclinic, *P*2_1_	Cu *K*α radiation, λ = 1.5418 Å
*a* = 9.3057 (6) Å	Cell parameters from 3289 reflections
*b* = 7.6934 (6) Å	θ = 3.4–72.8°
*c* = 13.4012 (11) Å	µ = 1.20 mm^−^^1^
β = 96.243 (7)°	*T* = 123 K
*V* = 953.74 (12) Å^3^	Platey fragment, colourless
*Z* = 4	0.30 × 0.20 × 0.04 mm

### Dimethyl (2S,3R)-3-Hydroxy-5-oxo-2,3,4,5-tetrahydrofuran-2,3-dicarboxylate (II) Data collection

**Table:**

Oxford Diffraction Gemini S CCD diffractometer	2976 reflections with *I* > 2σ(*I*)
Radiation source: sealed tube	*R*_int_ = 0.036
ω scans	θ_max_ = 73.4°, θ_min_ = 3.3°
Absorption correction: multi-scan (CrysAlis PRO; Oxford Diffraction, 2010)	*h* = −11→11
*T*_min_ = 0.747, *T*_max_ = 1.000	*k* = −8→9
8046 measured reflections	*l* = −16→14
3506 independent reflections	

### Dimethyl (2S,3R)-3-Hydroxy-5-oxo-2,3,4,5-tetrahydrofuran-2,3-dicarboxylate (II) Refinement

**Table:**

Refinement on *F*^2^	Hydrogen site location: mixed
Least-squares matrix: full	H atoms treated by a mixture of independent and constrained refinement
*R*[*F*^2^ > 2σ(*F*^2^)] = 0.044	*w* = 1/[σ^2^(*F*_o_^2^) + (0.0568*P*)^2^ + 0.1462*P*] where *P* = (*F*_o_^2^ + 2*F*_c_^2^)/3
*wR*(*F*^2^) = 0.121	(Δ/σ)_max_ < 0.001
*S* = 1.10	Δρ_max_ = 0.23 e Å^−^^3^
3506 reflections	Δρ_min_ = −0.22 e Å^−^^3^
281 parameters	Absolute structure: Flack *x* determined using 1098 quotients [(I+)-(I-)]/[(I+)+(I-)] (Parsons *et al.*, 2013)
3 restraints	Absolute structure parameter: 0.08 (17)

### Dimethyl (2S,3R)-3-Hydroxy-5-oxo-2,3,4,5-tetrahydrofuran-2,3-dicarboxylate (II) Special details

**Table:**

Geometry. All esds (except the esd in the dihedral angle between two l.s. planes) are estimated using the full covariance matrix. The cell esds are taken into account individually in the estimation of esds in distances, angles and torsion angles; correlations between esds in cell parameters are only used when they are defined by crystal symmetry. An approximate (isotropic) treatment of cell esds is used for estimating esds involving l.s. planes.

### Dimethyl (2S,3R)-3-Hydroxy-5-oxo-2,3,4,5-tetrahydrofuran-2,3-dicarboxylate (II) Fractional atomic coordinates and isotropic or equivalent isotropic displacement parameters (Å^2^)

**Table:**

	*x*	*y*	*z*	*U*_iso_*/*U*_eq_	
O1	0.0127 (3)	0.3894 (4)	0.4143 (2)	0.0393 (7)	
O2	−0.0438 (4)	0.4647 (5)	0.2547 (2)	0.0502 (8)	
O3	0.3364 (3)	0.3776 (4)	0.4119 (2)	0.0399 (7)	
H1H	0.407 (4)	0.349 (7)	0.458 (3)	0.048*	
O4	0.4351 (3)	0.0487 (4)	0.4527 (2)	0.0460 (8)	
O5	0.2148 (3)	−0.0633 (4)	0.4061 (2)	0.0432 (7)	
O6	0.1333 (3)	0.5084 (4)	0.5929 (2)	0.0460 (7)	
O7	0.2626 (3)	0.2625 (4)	0.6205 (2)	0.0431 (7)	
O8	0.5725 (3)	0.4161 (4)	−0.0594 (2)	0.0391 (7)	
O9	0.6406 (3)	0.5063 (5)	−0.2045 (2)	0.0484 (8)	
O10	0.8715 (3)	0.4023 (4)	0.0508 (2)	0.0388 (7)	
H2H	0.904 (5)	0.376 (7)	0.1128 (16)	0.047*	
O11	0.9307 (3)	0.0730 (4)	0.1173 (2)	0.0437 (7)	
O12	0.7678 (3)	−0.0374 (4)	−0.0028 (3)	0.0467 (8)	
O13	0.5379 (3)	0.5087 (4)	0.1312 (2)	0.0443 (7)	
O14	0.6548 (3)	0.2682 (4)	0.1911 (2)	0.0414 (7)	
C1	0.0312 (5)	0.3802 (6)	0.3161 (3)	0.0408 (9)	
C2	0.1501 (5)	0.2555 (7)	0.3003 (3)	0.0424 (10)	
H2A	0.2116	0.3012	0.2505	0.051*	
H2B	0.1106	0.1412	0.2771	0.051*	
C3	0.2350 (4)	0.2408 (6)	0.4034 (3)	0.0366 (9)	
C4	0.1125 (4)	0.2751 (6)	0.4721 (3)	0.0377 (9)	
H4	0.0637	0.1638	0.4870	0.045*	
C5	0.3083 (5)	0.0651 (6)	0.4238 (3)	0.0389 (9)	
C6	0.2722 (5)	−0.2380 (6)	0.4145 (4)	0.0477 (11)	
H6A	0.3464	−0.2521	0.3685	0.072*	
H6B	0.1940	−0.3216	0.3971	0.072*	
H6C	0.3151	−0.2587	0.4835	0.072*	
C7	0.1678 (4)	0.3672 (6)	0.5685 (3)	0.0378 (9)	
C8	0.3289 (6)	0.3351 (8)	0.7144 (4)	0.0564 (13)	
H8A	0.3960	0.4279	0.7003	0.085*	
H8B	0.3819	0.2437	0.7538	0.085*	
H8C	0.2537	0.3826	0.7524	0.085*	
C9	0.6617 (5)	0.4150 (6)	−0.1329 (3)	0.0393 (9)	
C10	0.7821 (4)	0.2878 (6)	−0.1067 (3)	0.0396 (9)	
H10A	0.8750	0.3340	−0.1252	0.048*	
H10B	0.7616	0.1755	−0.1414	0.048*	
C11	0.7864 (4)	0.2667 (6)	0.0067 (3)	0.0352 (9)	
C12	0.6230 (4)	0.2963 (6)	0.0191 (3)	0.0358 (9)	
H12	0.5694	0.1840	0.0090	0.043*	
C13	0.8388 (4)	0.0905 (6)	0.0475 (3)	0.0370 (9)	
C14	0.8023 (5)	−0.2154 (7)	0.0250 (4)	0.0478 (11)	
H14A	0.8702	−0.2623	−0.0192	0.072*	
H14B	0.7137	−0.2851	0.0182	0.072*	
H14C	0.8465	−0.2193	0.0947	0.072*	
C15	0.5978 (4)	0.3732 (6)	0.1191 (3)	0.0365 (9)	
C16	0.6380 (6)	0.3211 (7)	0.2932 (4)	0.0504 (12)	
H16A	0.6857	0.4334	0.3071	0.076*	
H16B	0.6820	0.2338	0.3402	0.076*	
H16C	0.5349	0.3319	0.3012	0.076*	

### Dimethyl (2S,3R)-3-Hydroxy-5-oxo-2,3,4,5-tetrahydrofuran-2,3-dicarboxylate (II) Atomic displacement parameters (Å^2^)

**Table:**

	*U*^11^	*U*^22^	*U*^33^	*U*^12^	*U*^13^	*U*^23^
O1	0.0391 (13)	0.0337 (18)	0.0436 (16)	0.0050 (13)	−0.0023 (11)	−0.0018 (13)
O2	0.0564 (18)	0.046 (2)	0.0456 (17)	0.0136 (15)	−0.0086 (14)	−0.0029 (14)
O3	0.0377 (14)	0.0332 (18)	0.0481 (16)	−0.0031 (13)	0.0012 (12)	0.0019 (13)
O4	0.0439 (16)	0.040 (2)	0.0519 (17)	0.0044 (14)	−0.0055 (13)	−0.0016 (13)
O5	0.0441 (16)	0.0288 (17)	0.0561 (18)	−0.0004 (13)	0.0023 (13)	0.0014 (13)
O6	0.0513 (16)	0.0371 (19)	0.0477 (17)	0.0081 (14)	−0.0031 (14)	−0.0090 (14)
O7	0.0528 (16)	0.0314 (18)	0.0429 (16)	0.0050 (14)	−0.0048 (13)	0.0029 (13)
O8	0.0386 (14)	0.0344 (18)	0.0431 (15)	0.0009 (12)	−0.0014 (11)	0.0040 (12)
O9	0.0510 (17)	0.049 (2)	0.0441 (17)	−0.0017 (15)	0.0000 (14)	0.0089 (14)
O10	0.0405 (14)	0.0341 (19)	0.0404 (15)	−0.0032 (13)	−0.0021 (11)	−0.0002 (12)
O11	0.0484 (16)	0.0373 (18)	0.0439 (16)	0.0042 (14)	−0.0015 (13)	−0.0001 (13)
O12	0.0433 (16)	0.0281 (19)	0.066 (2)	0.0007 (13)	−0.0051 (14)	−0.0063 (14)
O13	0.0493 (16)	0.039 (2)	0.0428 (16)	0.0079 (14)	−0.0027 (13)	−0.0032 (14)
O14	0.0492 (15)	0.0353 (18)	0.0400 (15)	0.0056 (14)	0.0063 (12)	0.0045 (13)
C1	0.044 (2)	0.033 (3)	0.043 (2)	0.0020 (19)	−0.0042 (17)	−0.0038 (18)
C2	0.049 (2)	0.036 (3)	0.040 (2)	0.0044 (19)	−0.0029 (17)	−0.0028 (18)
C3	0.041 (2)	0.027 (2)	0.041 (2)	−0.0035 (17)	0.0022 (16)	−0.0005 (16)
C4	0.0380 (19)	0.031 (2)	0.043 (2)	−0.0024 (17)	−0.0014 (16)	0.0003 (18)
C5	0.043 (2)	0.037 (3)	0.036 (2)	0.0028 (18)	0.0024 (16)	−0.0034 (17)
C6	0.055 (3)	0.032 (3)	0.056 (3)	0.002 (2)	0.005 (2)	0.002 (2)
C7	0.0351 (18)	0.039 (3)	0.039 (2)	−0.0013 (17)	0.0045 (15)	0.0006 (18)
C8	0.069 (3)	0.050 (3)	0.046 (3)	0.004 (2)	−0.015 (2)	0.002 (2)
C9	0.045 (2)	0.036 (3)	0.037 (2)	−0.0065 (18)	−0.0001 (16)	−0.0026 (17)
C10	0.041 (2)	0.037 (3)	0.040 (2)	−0.0018 (18)	0.0036 (16)	−0.0003 (17)
C11	0.0377 (19)	0.029 (2)	0.039 (2)	0.0002 (16)	0.0020 (15)	−0.0029 (17)
C12	0.039 (2)	0.026 (2)	0.042 (2)	−0.0024 (17)	−0.0012 (16)	0.0026 (17)
C13	0.0368 (19)	0.031 (2)	0.043 (2)	−0.0014 (17)	0.0050 (17)	−0.0033 (17)
C14	0.042 (2)	0.036 (3)	0.065 (3)	0.000 (2)	0.007 (2)	−0.004 (2)
C15	0.0339 (17)	0.031 (2)	0.044 (2)	−0.0025 (17)	0.0017 (15)	0.0001 (17)
C16	0.060 (3)	0.048 (3)	0.043 (2)	0.005 (2)	0.006 (2)	0.008 (2)

### Dimethyl (2S,3R)-3-Hydroxy-5-oxo-2,3,4,5-tetrahydrofuran-2,3-dicarboxylate (II) Geometric parameters (Å, º)

**Table:**

O1—C1	1.347 (5)	C2—H2B	0.9900
O1—C4	1.442 (5)	C3—C5	1.526 (6)
O2—C1	1.210 (5)	C3—C4	1.562 (6)
O3—C3	1.410 (5)	C4—C7	1.514 (6)
O3—H1H	0.880 (14)	C4—H4	1.0000
O4—C5	1.208 (5)	C6—H6A	0.9800
O5—C5	1.320 (5)	C6—H6B	0.9800
O5—C6	1.446 (6)	C6—H6C	0.9800
O6—C7	1.189 (6)	C8—H8A	0.9800
O7—C7	1.334 (5)	C8—H8B	0.9800
O7—C8	1.451 (6)	C8—H8C	0.9800
O8—C9	1.355 (5)	C9—C10	1.501 (6)
O8—C12	1.439 (5)	C10—C11	1.524 (6)
O9—C9	1.188 (5)	C10—H10A	0.9900
O10—C11	1.401 (5)	C10—H10B	0.9900
O10—H2H	0.876 (14)	C11—C13	1.522 (6)
O11—C13	1.204 (5)	C11—C12	1.565 (5)
O12—C13	1.328 (5)	C12—C15	1.506 (6)
O12—C14	1.446 (6)	C12—H12	1.0000
O13—C15	1.202 (5)	C14—H14A	0.9800
O14—C15	1.324 (5)	C14—H14B	0.9800
O14—C16	1.452 (6)	C14—H14C	0.9800
C1—C2	1.497 (6)	C16—H16A	0.9800
C2—C3	1.519 (6)	C16—H16B	0.9800
C2—H2A	0.9900	C16—H16C	0.9800
			
C1—O1—C4	110.4 (3)	H8A—C8—H8B	109.5
C3—O3—H1H	108 (4)	O7—C8—H8C	109.5
C5—O5—C6	116.8 (3)	H8A—C8—H8C	109.5
C7—O7—C8	114.6 (4)	H8B—C8—H8C	109.5
C9—O8—C12	110.5 (3)	O9—C9—O8	121.5 (4)
C11—O10—H2H	110 (4)	O9—C9—C10	128.9 (4)
C13—O12—C14	119.2 (4)	O8—C9—C10	109.6 (4)
C15—O14—C16	116.1 (4)	C9—C10—C11	103.9 (3)
O2—C1—O1	120.7 (4)	C9—C10—H10A	111.0
O2—C1—C2	129.0 (4)	C11—C10—H10A	111.0
O1—C1—C2	110.3 (4)	C9—C10—H10B	111.0
C1—C2—C3	103.7 (3)	C11—C10—H10B	111.0
C1—C2—H2A	111.0	H10A—C10—H10B	109.0
C3—C2—H2A	111.0	O10—C11—C13	111.5 (3)
C1—C2—H2B	111.0	O10—C11—C10	107.1 (3)
C3—C2—H2B	111.0	C13—C11—C10	115.2 (4)
H2A—C2—H2B	109.0	O10—C11—C12	111.0 (3)
O3—C3—C2	107.1 (3)	C13—C11—C12	111.6 (3)
O3—C3—C5	111.4 (3)	C10—C11—C12	99.8 (3)
C2—C3—C5	114.0 (4)	O8—C12—C15	109.2 (3)
O3—C3—C4	110.6 (4)	O8—C12—C11	105.0 (3)
C2—C3—C4	100.6 (3)	C15—C12—C11	113.5 (3)
C5—C3—C4	112.6 (4)	O8—C12—H12	109.6
O1—C4—C7	108.2 (3)	C15—C12—H12	109.6
O1—C4—C3	104.8 (3)	C11—C12—H12	109.6
C7—C4—C3	112.3 (3)	O11—C13—O12	125.7 (4)
O1—C4—H4	110.5	O11—C13—C11	123.5 (4)
C7—C4—H4	110.5	O12—C13—C11	110.8 (3)
C3—C4—H4	110.5	O12—C14—H14A	109.5
O4—C5—O5	125.5 (4)	O12—C14—H14B	109.5
O4—C5—C3	123.5 (4)	H14A—C14—H14B	109.5
O5—C5—C3	111.0 (3)	O12—C14—H14C	109.5
O5—C6—H6A	109.5	H14A—C14—H14C	109.5
O5—C6—H6B	109.5	H14B—C14—H14C	109.5
H6A—C6—H6B	109.5	O13—C15—O14	125.9 (4)
O5—C6—H6C	109.5	O13—C15—C12	125.4 (4)
H6A—C6—H6C	109.5	O14—C15—C12	108.6 (4)
H6B—C6—H6C	109.5	O14—C16—H16A	109.5
O6—C7—O7	126.2 (4)	O14—C16—H16B	109.5
O6—C7—C4	125.9 (4)	H16A—C16—H16B	109.5
O7—C7—C4	107.9 (4)	O14—C16—H16C	109.5
O7—C8—H8A	109.5	H16A—C16—H16C	109.5
O7—C8—H8B	109.5	H16B—C16—H16C	109.5
			
C4—O1—C1—O2	179.0 (4)	C12—O8—C9—O9	−179.1 (4)
C4—O1—C1—C2	−0.5 (5)	C12—O8—C9—C10	0.3 (5)
O2—C1—C2—C3	160.9 (5)	O9—C9—C10—C11	158.1 (5)
O1—C1—C2—C3	−19.7 (5)	O8—C9—C10—C11	−21.2 (5)
C1—C2—C3—O3	−86.2 (4)	C9—C10—C11—O10	−84.8 (4)
C1—C2—C3—C5	150.1 (4)	C9—C10—C11—C13	150.5 (4)
C1—C2—C3—C4	29.3 (5)	C9—C10—C11—C12	30.9 (4)
C1—O1—C4—C7	139.9 (4)	C9—O8—C12—C15	142.4 (3)
C1—O1—C4—C3	19.9 (4)	C9—O8—C12—C11	20.3 (4)
O3—C3—C4—O1	82.7 (4)	O10—C11—C12—O8	81.3 (4)
C2—C3—C4—O1	−30.2 (4)	C13—C11—C12—O8	−153.6 (3)
C5—C3—C4—O1	−152.0 (3)	C10—C11—C12—O8	−31.4 (4)
O3—C3—C4—C7	−34.5 (5)	O10—C11—C12—C15	−37.9 (5)
C2—C3—C4—C7	−147.5 (4)	C13—C11—C12—C15	87.1 (4)
C5—C3—C4—C7	90.8 (4)	C10—C11—C12—C15	−150.6 (4)
C6—O5—C5—O4	−5.2 (6)	C14—O12—C13—O11	−1.2 (7)
C6—O5—C5—C3	175.1 (4)	C14—O12—C13—C11	180.0 (4)
O3—C3—C5—O4	6.5 (6)	O10—C11—C13—O11	8.2 (6)
C2—C3—C5—O4	127.9 (5)	C10—C11—C13—O11	130.5 (4)
C4—C3—C5—O4	−118.4 (5)	C12—C11—C13—O11	−116.6 (4)
O3—C3—C5—O5	−173.8 (3)	O10—C11—C13—O12	−173.0 (3)
C2—C3—C5—O5	−52.4 (5)	C10—C11—C13—O12	−50.6 (5)
C4—C3—C5—O5	61.3 (5)	C12—C11—C13—O12	62.3 (4)
C8—O7—C7—O6	−1.3 (6)	C16—O14—C15—O13	2.1 (6)
C8—O7—C7—C4	178.3 (4)	C16—O14—C15—C12	−178.9 (4)
O1—C4—C7—O6	1.0 (6)	O8—C12—C15—O13	3.7 (5)
C3—C4—C7—O6	116.1 (5)	C11—C12—C15—O13	120.6 (5)
O1—C4—C7—O7	−178.6 (3)	O8—C12—C15—O14	−175.2 (3)
C3—C4—C7—O7	−63.4 (4)	C11—C12—C15—O14	−58.4 (4)

### Dimethyl (2S,3R)-3-Hydroxy-5-oxo-2,3,4,5-tetrahydrofuran-2,3-dicarboxylate (II) Hydrogen-bond geometry (Å, º)

**Table:**

*D*—H···*A*	*D*—H	H···*A*	*D*···*A*	*D*—H···*A*
O3—H1*H*···O4^i^	0.88 (1)	2.36 (5)	2.951 (4)	125 (4)
O10—H2*H*···O2^ii^	0.88 (1)	2.03 (3)	2.802 (4)	147 (5)

Symmetry codes: (i) −*x*+1, *y*+1/2, −*z*+1; (ii) *x*+1, *y*, *z*.
